# Mycotoxigenic Fungi and Natural Co-Occurrence of Mycotoxins in Rainbow Trout (*Oncorhynchus mykiss*) Feeds

**DOI:** 10.3390/toxins7114595

**Published:** 2015-11-05

**Authors:** Mariana Greco, Alejandro Pardo, Graciela Pose

**Affiliations:** 1 Laboratorio de Micología Molecular, Departamento de Ciencia y Tecnología, Universidad Nacional de Quilmes (UNQ), Roque Sáenz Peña 352, Bernal, Buenos Aires 1876, Argentina; E-Mail: apardo@unq.edu.ar; 2 Consejo Nacional de Investigaciones Científicas y Técnicas (CONICET), Avenida Rivadavia 1917, Buenos Aires 1033, Argentina; 3 Escuela de Producción, Tecnología y Medio Ambiente, Universidad Nacional de Río Negro (UNRN), Mitre 331, Villa Regina 8336, Río Negro, Argentina; E-Mail: npose@unrn.edu.ar

**Keywords:** aquaculture, fish feed, *Oncorhynchus mykiss*, mycobiota, mycotoxins

## Abstract

Samples of rainbow trout feed were analyzed with the aim to determine the mycobiota composition and the co-occurrence of mycotoxins. A total of 28 samples of finished rainbow trout feed from hatcheries in the provinces of Río Negro and Neuquén, Argentina, were studied. Fungal counts were obtained on three culture media in the ranges of <10 to 4.2 × 10^4^ CFU/g on Dichloran Rose Bengal Chloramphenicol Agar (DRBC), <10 to 5.1 × 10^4^ CFU/g on Dichloran Chloramphenicol Peptone Agar (DCPA) and <10 to 3.6 × 10^4^ CFU/g on Dichloran 18% Glycerol Agar (DG18). The most frequent mycotoxigenic fungi were *Eurotium* (frequency (Fr) 25.0%), followed by *Penicillium* (Fr 21.4%) and *Aspergillus* (Fr 3.6%). The most prevalent mycotoxigenic species were *E. repens* (Fr 21.4%) and *E. rubrum* (Fr 14.3%). All samples were contaminated with mycotoxins: 64% samples were contaminated with T-2 toxin (median 70.08 ppb), 50% samples with zearalenone (median 87.97 ppb) and aflatoxins (median 2.82 ppb), 25% with ochratoxin A (median 5.26 ppb) and 3.57% samples with deoxynivalenol (median 230 ppb). Eight samples had a fumonisins contamination level below the limit of detection. Co-occurrence of six mycotoxins was determined in 7% of the samples.

## 1. Introduction

Rainbow trout (*Oncorhynchus mykiss*) production has been growing exponentially for the last 50 years in Europe and Chile, the latter being the largest producer [1]. In Argentina, commercial aquaculture activity began to expand in the 1990s, mostly in the provinces of Río Negro and Neuquén, reaching in 2012 a rainbow trout crop of 1260 tonnes, which represents 42% of the national aquatic production [2]. About ninety percent of the United States’ imported rainbow trout comes from Chile, Canada and Argentina [3].

Trout products for human consumption are commercially available as fresh, filleted, smoked, canned, whole and frozen, among others [1,3], for restaurants, supermarkets or consumers [3]. Fish and seafood are present in many dietary guidelines, since their consumption is known to have positive health effects [4,5,6].

Feed for rainbow trout includes soybean expeller, disabled soybean, corn, wheat, wheat bran, corn gluten meal, soybean oil, fish meal and fish oil, among others, formulated for different life stages and presented as compact pellets and in different sizes. Current trout production is exclusively based on commercial feed [7]. As a consequence, this general practice can lead to increased cereal mycotoxin concentrations in fish feed [8]. Furthermore, the presence of molds in fish food is indicative of contamination probably due to an inappropriate selection of ingredients for manufacturing or improper storage as potential sources of mycotoxins. The intake of low quality feeds can have adverse effects on animal health and productivity [9,10,11]. Moreover, if mycotoxins are carried over into the meat and eggs of the farmed fish, the contaminated feed may pose an additional health risk to the consumers [12].

Among the genera of fungi recognized to be toxigenic, most species belong to *Aspergillus*, *Fusarium*, *Penicillium* and *Alternaria*. Additionally, the most relevant mycotoxins to animal production, based on their toxicity and occurrence, are aflatoxins, zearalenone, T-2 toxin, deoxynivalenol, ochratoxin A, fumonisins and patulin.

Aflatoxicosis outbreak cases in fish have been reported in the United States [13,14], Germany [15], Mexico [16] and Denmark [17]. Rainbow trout is extremely sensitive to aflatoxin B1 (AFB1) [18,19,20,21], causing hepatic damage [22], anemia, hemorrhage, liver damage, weight loss, increased susceptibility to secondary infectious diseases and mortality [23]. Acute aflatoxicosis causes liver failure, and chronic exposure provokes immunosuppression [24].

Ochratoxin A (OTA) has been shown to be nephrotoxic, carcinogenic, teratogenic and to have immunosuppressive effects and has been widely reported as a contaminant in animal feeds in the livestock industry, mainly in poultry feeds [25,26,27,28]. Doster *et al*. [29] reported OTA intoxication effects on rainbow trout, which cause kidney and liver necrosis and finally death.

Presence of *Fusarium* mycotoxins, trichothecenes (deoxynivalenol (DON) and T-2 toxin), fumonisins (FUM) and zearalenone (ZEA) in contaminated fish feeds can cause adverse effects: trichothecenes cause reduced feed intake and growth rates [30], performance reduction, immune impairment and organ lesions [31,32,33]. Zearalenone can have estrogenic effects, and fumonisins can cause reduce growth and increased liver glycogen [34]. Rainbow trout is also very sensitive to DON present in naturally-contaminated grains [35]. Fumonisins are found in corn grain, which is a major component of aquacultural feeds for warm water fish [5]. Fumonisin toxicity disrupts sphingolipid metabolism [24], which provokes abnormal higher levels of sphinganine accumulation in different tissues, including the liver [5]. Fumonisin B1 promotes aflatoxin B1 and *N*-methyl-*N*′-nitro-nitrosoguanidine-initiated liver tumors in rainbow trout [36]. The T-2 toxin reduces feed consumption and growth and lowers the hematocrit and blood hemoglobin in rainbow trout at levels higher than 2.5 ppm. T-2 toxin levels above 10 ppm have caused gastrointestinal bleeding and regurgitation in adult trout [37].

There are previous works reporting the presence of one or two toxins, in particular in commercial fish feed or fish organs [8,12,19,21,38,39,40]. However, recent studies and literature reviews have shown that different kinds of finished animal feeds were co-contaminated with several different toxins, which is the more likely situation [9,10,41,42,43,44,45]. Thus, in order to have a complete risk profile of the feedstuff, it is not enough to study the occurrence of one mycotoxin alone [43].

The aim of this work was to study and determine the toxicogenic mycobiota, including enumeration and identification of mold genera and species, and quantification of the major mycotoxins. To our knowledge, this is the first report in Argentina documenting the presence of molds and the natural occurrence of the six most important mycotoxins in commercial rainbow trout feed.

## 2. Results

### 2.1. Mycobiota Analysis

Fungal counts (CFU/g) were obtained from different rainbow trout feed samples on the three culture media. On Dichloran Rose Bengal Chloramphenicol Agar (DRBC), the fungal count range was <10 to 4.2 × 10^4^ CFU/g; on Dichloran Chloramphenicol Peptone Agar (DCPA), the range was <10 to 5.1 × 10^4^ CFU/g. Xerophilic fungal counts were in the range <10 to 3.6 × 10^4^ CFU/g. The fungal counts’ average and median were similar between all culture media tested (103 CFU/g and 102 CFU/g, respectively). Non-significant differences in fungal count were found between feed type, brands and hatcheries.

Mold genera recovered in this study, as well as frequency (Fr%) and relative density (RD%) are shown in Table 1. Three mold genera known to be mycotoxigenic were recorded [46]. The most prevalent mycotoxigenic fungi were the *Eurotium* genus (recovered from seven samples), followed by *Penicillium* (six samples) and *Aspergillus* (one sample). *Cladosporium*, *Trichoderma* and other mitosporic Ascomycetes were also found. One genus belonging to *Zygomycetes*, *Mucor*, was determined, as well as high levels of yeast contamination.

A total of 13 species were recovered from rainbow trout feed samples. The frequency (Fr%) and relative density (RD%) are shown in Table 2. According to the isolation frequency (Fr%) and relative density (RD%), *E. repens* and *E. rubrum* were the most prevalent mycotoxigenic fungi present (Fr: 21.4% and 14.3%; RD: 15.8% and 10.5%, respectively).

Table 3 provides information about the mycobiota composition in each sample. From this table, the genus and species distribution, as well as mycotoxins present in each sample can be observed.

**Table 1:** Fungal genera present in rainbow trout feeds.

Genus	Number of occurrences	Fr (%) ^1^	RD (%) ^2^
*Aspergillus*	1	3.6	2.0
*Cladosporium*	15	53.6	30.0
*Eurotium*	7	25.0	14.0
*Mucor*	4	14.3	8.0
*Penicillium*	6	21.4	12.0
*Trichoderma*	1	3.6	2.0
Yeast	12	42.9	24.0
Others	4	14.3	8.0

^1^ Isolation frequency; ^2^ isolation relative density.

**Table 2:** Fungal species present in rainbow trout feed samples.

Species	Number of occurrences	Fr (%) ^1^	RD (%) ^2^
*Aspergillus versicolor*	1	3.6	2.6
*Cladosporium cladosporioides*	15	53.6	39.5
*C. herbarum*	1	3.6	2.6
*Eurotium repens*	6	21.4	15.8
*E. rubrum*	4	14.3	10.5
*Eurotium* sp.	1	3.6	2.6
*Mucor* sp.	2	7.1	5.3
*Penicillium chrysogenum*	1	3.6	2.6
*P. corylophilum*	1	3.6	2.6
*P. crustosum*	1	3.6	2.6
*P. expansum*	1	3.6	2.6
*P. nalgiovense*	3	10.7	7.9
*Trichoderma harzianum*	1	3.6	2.6

^1^ Isolation frequency; ^2^ isolation relative density.

### 2.2. Mycotoxin Analysis

We determined that all samples were contaminated with mycotoxins. Table 3 shows the mycotoxin distribution in each sample; from this table, it can be observed that not necessarily the presence of a particular toxin was correlated with the presence of the possible toxin producer mold. Table 4 shows the concentration values of each mycotoxin in the samples. Contamination with mycotoxins was detected in all of the samples analyzed: aflatoxins (AFs), median 2.82 ppb; deoxynivalenol (DON), median 230 ppb; ochratoxin A (OTA), median 5.26 ppb; T-2 toxin, median 70.08 ppb; zearalenone (ZEA), median 87.97 ppb. No significant differences were found for total count in the different provinces and growth stage (*p* = 0.8615). Neither were significant differences determined for toxins in the provinces and growth stage (AFs: *p* = 0.1799; DON: *p* = 0.3927; FUM: *p* = 0.6687; OTA: *p* = 0.2778; T-2 toxin: *p* = 0.0852; ZEA: *p* = 0.2583). The Spearman’s rank correlation test did not show a correlation between the studied mycotoxins.

**Table 3:** Genus, species and mycotoxin distribution in the samples.

Sample	Genus Profile	Species Profile	Mycotoxin Profile					
1	*Cladosporium*	*C. cladosporioides*	*---*	*---*	*---*	OTA	T-2	*---*
	yeast	*---*						
2	*Cladosporium*	*C. cladosporioides*	*---*	*---*	*---*	OTA ^*^	T-2	*---*
3	*Aspergillus*	*A. versicolor*	*---*	DON ^*^	*---*	OTA	T-2	*---*
	*Cladosporium*	*C. cladosporioides*						
	yeast, others	*---*						
4	*Cladosporium*	*C. cladosporioides*	AFs ^*^	DON ^*^	FUM ^*^	*---*	T-2	ZEA
5	*Cladosporium*	*C. cladosporioides*	---	DON ^*^	FUM ^*^	OTA	T-2	ZEA
6	*Cladosporium*	*C. cladosporioides*	AFs	DON ^*^	FUM ^*^	*---*	T-2	ZEA
	yeast	*---*						
7	*Cladosporium Trichoderma*	*C. cladosporioides*	AFs	DON ^*^	FUM ^*^	OTA ^*^	T-2	ZEA
		*T. harzianum*						
8	*Cladosporium*	*C. cladosporioides*	AFs	DON ^*^	FUM ^*^	*---*	T-2	ZEA
	yeast, others	*---*						
9	*Cladosporium*	*C. cladosporioides*	AFs ^*^	DON ^*^	FUM ^*^	*---*	T-2	ZEA
	others	*---*						
10	*Mucor*	*Mucor* sp.	*---*	DON ^*^	*---*	OTA	T-2	ZEA ^*^
	*Penicillium*	*P. crustosum*						
		*P. corylophilum*						
11	*---*	---	*-*--	DON ^*^	--*-*	OTA	T-2	ZEA
12	*Cladosporium*	*C. cladosporioides*	AFs	DON ^*^	*---*	OTA ^*^	T-2 ^*^	ZEA ^*^
	*Penicillium*	*C. herbarum*						
	yeast, others	*P. expansum*						
13	*Cladosporium*	*C. cladosporioides*	AFs	DON ^*^	*---*	OTA ^*^	T-2 ^*^	*---*
	yeast	*---*						
14	Yeast	*---*	AFs	*---*	*---*	OTA ^*^	T-2	ZEA ^*^
15	*Cladosporium*	*C. cladosporioides*	AFs ^*^	*---*	*---*	OTA	T-2	ZEA ^*^
16	*Mucor*	*Mucor* sp.	AFs	*---*	FUM ^*^	OTA	T-2	ZEA ^*^
17	Yeast	*---*	AFs	DON ^*^	FUM ^*^	OTA ^*^	T-2	ZEA ^*^
18	*Penicillium*	*P. nalgiovense*	AFs	DON ^*^	*---*	*---*	T-2	ZEA ^*^
19	*Cladosporium*	*C. cladosporioides*	AFs	DON	*---*	*---*	T-2	ZEA ^*^
	*Eurotium*	*E. repens*						
	*Mucor*	*Mucor* sp.						
	yeast	*---*						
20	*Cladosporium*	*C. cladosporioides*	AFs	DON ^*^	*---*	*---*	T-2	ZEA ^*^
	*Penicillium*	*P. nalgiovense*						
	yeast	*---*						
21	*Eurotium*	*E. rubrum*	*---*	*---*	*---*	OTA ^*^	T-2 ^*^	ZEA ^*^
	yeast	*---*						
22	*Mucor*	*Mucor* sp.	AFs ^*^	*---*	*---*	OTA ^*^	*---*	ZEA
	*Penicillium*	*P. nalgiovense*						
23	Yeast	*---*	*---*	*---*	*---*	*---*	*---*	ZEA
24	*Cladosporium*	*C. cladosporioides*	*---*	*---*	*---*	*---*	*---*	ZEA
	*Eurotium*	*E. repens*, *E. rubrum*						
	*Penicillium*	*P. chrysogenum*						
25	*Eurotium*	*E. repens, Eurotium* sp.	AFs	*---*	*---*	*---*	*---*	ZEA
26	*Eurotium*	*E. repens*	AFs	*---*	*---*	---	*---*	ZEA
27	*Eurotium*	*E. repens*, *E. rubrum*	*---*	*---*	*---*	OTA ^*^	*---*	ZEA
28	*Eurotium*	*E. repens*, *E. rubrum*	AFs	*---*	*---*	*---*	*---*	ZEA

AFs: aflatoxins; DON: deoxynivalenol; FUM: fumonisins; OTA: ochratoxin A; T-2: T-2 toxin; ZEA: zearalenone; ^*^ contamination observed below the value set as the LOD (limit of detection).

**Table 4:** Co-occurrence of mycotoxins in rainbow trout feed samples (ND: not detected).

Sample	Mycotoxins (ppb)											
	AFs		DON		FUM		OTA		T-2		ZEA	
1	<1.7 ^a^	0 ^b^	<222 ^a^	0 ^b^	<222 ª	0 ^b^	5.23	-	68.03	-	<50	0 ^b^
2	<1.7 ^a^	0 ^b^	<222 ^a^	0 ^b^	<222 ª	0 ^b^	<5 ^a^	3.5 ^c^	75.99	-	<50	0 ^b^
3	<1.7 ^a^	0 ^b^	<222 ^a^	165 ^c^	<222 ª	0 ^b^	5.26	-	83.87	-	<50	0 ^b^
4	<1.7 ^a^	1.3 ^c^	<222 ^a^	178 ^c^	<222 ª	190 ^c^	<5 ^a^	0 ^b^	62.57	-	67.98	-
5	<1.7 ^a^	0 ^b^	<222 ^a^	207 ^c^	<222 ª	205 ^c^	8.79	-	60.95	-	56.91	-
6	2.7	-	<222 ^a^	195 ^c^	<222 ª	209 ^c^	<5 ^a^	0 ^b^	104.44	-	95.53	-
7	1.78	-	<222 ^a^	179 ^c^	<222 ª	222 ^c^	<5 ^a^	4.9 ^c^	105.99	-	88.7	-
8	1.97	-	<222 ^a^	169 ^c^	<222 ª	208 ^c^	<5 ^a^	0 ^b^	104.22	-	52.65	-
9	<1.7 ^a^	1.5 ^c^	<222 ^a^	155 ^c^	<222 ª	222 ^c^	<5 ^a^	0 ^b^	63.71	-	67.3	-
10	<1.7 ^a^	0 ^b^	<222 ^a^	184 ^c^	ND	0	5.14	-	75.35	-	<50	32.0 ^c^
11	<1.7 ^a^	0 ^b^	<222 ^a^	156 ^c^	<222 ^a^	0 ^b^	5.52	-	102.69	-	62.83	-
12	2.58	-	<222 ^a^	210 ^c^	<222 ª	0 ^b^	<5 ^a^	3.7 ^c^	<50	50 ^c^	<50	20.4 ^c^
13	2.79	-	<222 ^a^	150 ^c^	<222 ª	0 ^b^	<5 ^a^	4.1 ^c^	<50	50 ^c^	<50	0 ^b^
14	2.97	-	<222 ^a^	0 ^b^	<222 ª	0 ^b^	<5 ^a^	3.5 ^c^	57.16	-	<50	22.6 ^c^
15	<1.7 ^a^	1.7 ^c^	<222 ^a^	0 ^b^	<222 ª	0 ^b^	6.28	-	60.87	-	<50	35.0 ^c^
16	4.19	-	<222 ^a^	0 ^b^	<222 ª	191 ^c^	5.23	-	75.23	-	<50	32.6 ^c^
17	3.87	-	<222 ^a^	205 ^c^	<222 ª	187 ^c^	<5 ^a^	3.6 ^c^	71.05	-	<50	42.1 ^c^
18	2.85	-	<222 ^a^	164 ^c^	<222 ª	0 ^b^	<5 ^a^	0 ^b^	57.44	-	<50	33.7 ^c^
19	3.09	-	230	-	<222 ª	0 ^b^	<5 ^a^	0 ^b^	60.00	-	<50	39.5 ^c^
20	2.77	-	<222 ^a^	205 ^c^	<222 ª	0 ^b^	<5 ^a^	0 ^b^	69.11	-	<50	24.3 ^c^
21	ND	0	<222 ^a^	0 ^b^	<222 ª	0 ^b^	<5 ^a^	5.0 ^c^	<50	50 ^c^	<50	50.0 ^c^
22	<1.7 ^a^	1.7 ^c^	<222 ^a^	0 ^b^	<222 ª	0 ^b^	<5 ^a^	4.8 ^c^	ND	0	147.45	-
23	ND	0	<222 ^a^	0 ^b^	<222 ª	0 ^b^	<5 ^a^	0 ^b^	ND	0	159.76	-
24	ND	0	<222 ^a^	0 ^b^	<222 ª	0 ^b^	<5 ^a^	0 ^b^	ND	0	102.81	-
25	1.87	-	<222 ^a^	0 ^b^	<222 ª	0 ^b^	<5 ^a^	0 ^b^	ND	0	95.77	-
26	7.05	-	<222 ^a^	0 ^b^	<222 ª	0 ^b^	<5 ^a^	0 ^b^	ND	0	71.53	-
27	ND	0	<222 ^a^	0 ^b^	<222 ª	0 ^b^	<5 ^a^	5.0 ^c^	ND	0	110.26	-
28	8.91	-	<222 ^a^	0 ^b^	ND	0	<5 ^a^	0 ^b^	ND	0	87.24	-
Median ^d^	2.82		230		---		5.26		70.08		87.97	
LOD *	<1.7		222		222		5		<20		29	
LOQ *^,+^	---		222		---		---		50		50	

* ppb (according to the manufacturer’s provided information); ^+^ limit of quantification; ^a^ detected below the LOD (limit of detection); ^b^ estimated concentrations below LOD/√2 were assumed to be zero; ^c^ concentrations calculated by estimation with the Rida Soft win software; for statistical purposes results >LOD/√2 were considered [47]; ^d^ median of positive excludes results of estimated concentrations; - values above the LOD were not estimated.

Co-occurrence of at least two out of six mycotoxins was recorded in 93% (26/28) of samples analyzed. Co-occurrence of six mycotoxins was determined in 7% (2/28) of the samples.

## 3. Discussion

The raw material used in animal feed production is usually the source of molds and mycotoxins [48]. In this study, it was found that 26 out of 28 samples analyzed were contaminated with fungi, whereas all of the samples with mycotoxins.

Moderate levels of fungal counts were determined on DRBC (<10 to 4.2 × 10^4^ UFC/g) and DG18 (xerophilic fungi) (<10 to 3.6 × 10^4^ UFC/g). Similar results were reported in Brazil on finished fish feeds [40,45]. However, Nunes [49] reported values lower than 1 × 10^7^ UFC/g on ration formulated for fish feed. Regarding fungal count, 1 × 10^4^ UFC/g on DRBC was proposed as the limit of hygienic feed quality [50]. According to our study, 10.7% of the samples were not in agreement with the limit. Barbosa *et al*. [45] also reported that 10% of the samples analyzed by them exceeded the limit of hygienic quality proposed. Cardoso [40] reported 56% of juvenile stage and 11% growing stages samples exceeding the limit of hygienic quality, and Nunes [49] reported 67% of samples exceeding it. High fungal counts would indicate poor quality of raw materials and/or poor manufacturing practices. Fungal growth in raw material affects the nutritional feed quality [10]. Feeds that do not meet the criteria of hygienic quality are a risk to animal health.

There are a few studies reporting mycobiota on finished fish feed [39,40,45] and even less on rainbow trout feeds [21]. In our study, predominant mycotoxigenic genera were *Eurotium* (Fr 25.00%) and *Penicillium* (Fr 21.43%). The most prevalent mycotoxigenic species were *E. repens* (21.43%) and *E. rubrum* (14.29%). This result differs from those obtained by Alinezhad *et al*. [21] on rainbow trout feed, who found the *Aspergillus* genus as the most prevalent (57.0%), followed by *Penicillium* (12.84%) and *A. flavus* (60.66%) as the most prevalent species. In Brazil, studies conducted on aquaculture feeds have also shown the presence of species belonging to *Aspergillus* and *Penicillium* genera. Cardoso [40] reported an occurrence of 56.16% of *Aspergillus* and teleomorphs in rations for fish, followed by *Penicillium* (19.18%), *Cladosporium* (16.44%) and *Fusarium* (8.22%), *A. flavus* (60%) being the most common species, followed by *Eurotium* sp. Barbosa *et al*. [45] studied the mycobiota and mycotoxin content of finished feed samples from tilapia farms and did not find species belonging to *Fusarium* genera. *Aspergillus* was the most prevalent toxicogenic genera (68%) followed by *Penicillium* and *A. niger* aggregate, *A. flavu*s being the most prevalent species [45]. In Portugal, Almeida *et al*. [39] have also reported the presence of *Aspergillus*, *Penicillium*, *Cladosporium* and *Fusarium* from feed samples for farmed sea bass. Again, *A. flavus* was the most frequent mold found on 40.2% of the samples tested.

With respect to mycotoxigenic fungi, several studies have been done regarding the toxicity of *Aspergillus* and *Fusarium* mycotoxins on fish [22,23,29,30,31,32,33,34,35]. Considering the frequent presence of *Eurotium* and *Penicillium* species (although found in less proportion), determined in our work and reported by other authors [40,45], further studies should be done regarding the toxicity of *Eurotium* and *Penicillium* mycotoxins in fish and its occurrence in finished fish feeds. *Eurotium* and *Penicillium* species could also be able to produce a wide range of toxic compounds. However, there is no information about the toxicity of these compounds in fish [45] or its occurrence in finished fish feeds.

Regarding animal feed, the EU establishes regulations/recommendations for several mycotoxins. Although is not common to observe an excess of those limits [42], a low level of contaminations and co-contamination is frequent [9,10,41,42,43,44,51]. According to this, in our work, a total of six mycotoxins were detected in the samples tested. Our values were between <1.7 to 4.19 ppb. Co-occurrence of at least two out of six mycotoxins was recorded in 93% of the samples of finished rainbow trout feeds analyzed from hatcheries in Argentina.

In another study on fish feed, pellets of rainbow trout feed and feed ingredients were tested for AFB1, and except gluten, all of them were contaminated with aflatoxins in the range of 0.06 to 212.18 ppb [21]. The occurrence of DON and ZEA was determined in samples of commercial fish feed collected from central Europe. A high percentage (80%) of commercial fish feed samples were found to be contaminated with DON at averages values of 289 ppb, and ZEA was found in all samples, showing an average value of 67.9 ppb [51]. Barbosa *et al*. [45] detected FB1 in 90% of finished fish feed samples analyzed with an average value of 2.6 ppb. Furthermore, Barbosa *et al*. [45] found AFB1 and OTA contaminated samples at no quantifiable levels. The intake of low levels of mycotoxins may lead to the deterioration of the immune system, causing animal health problems and economic losses due to a decrease in productivity [48].

Simultaneous occurrence of mycotoxins was also determined by other researchers. DON and ZEA were determined in nine out 11 samples of commercial fish feed in central Europe [51]. Furthermore, Barbosa *et al.* [45] determined the co-occurrence of the carcinogenic mycotoxins AFB1, OTA and FB1 in finished fish feeds from farms in Brazil. Streit *et al*. [44] performed a multi-mycotoxin screening on 83 samples of feed and feed ingredients from Europe, revealing the occurrence of 139 different secondary metabolites, and all of the samples analyzed contained seven to 69 metabolites, showing a high number of co-occurring metabolites. A review by Streit *et al*. [43] revealed that 75% to 100% of the samples of animal feeds contained more than one mycotoxin, which even at low doses are able to cause animal health problems. In South Africa, 92 samples of different animal feed types were analyzed for 11 mycotoxins (aflatoxins, ochratoxin A, fumonisins, zearalenone and trichothecenes), and except for a low number of samples, the levels of detected mycotoxins could be considered safe according to South African legislation [42]. However, mycotoxin co-occurrence was common [42]. Synergistic effects of simultaneous exposure of animals to more than one toxin could be expected as able to cause mycotoxicoses [36,42,52].

In our case, the presence of a particular toxin was not necessarily correlated with the presence of the possible toxin producer mold (Table 3). These results were previously observed in finished fish feeds [45]. Fungal growth and mycotoxin production on crops are influenced by several factors [52]. Particularly, *Fusarium* is a field fungi usually lacking the ability to grow on dry feed. However, it is possible that mycotoxins produced by the fungus in the field come with the raw material that composes the finished feed [45]. The other way around, the presence of mycotoxin-producing fungi in feedstuffs is not always conducive to contamination with mycotoxins. Besides, taking into account that the conditions of the feed manufacturing process involve feed exposure to high temperatures, fungal propagules inactivation may occur, but not for mycotoxins, since they are heat stable [45,46,53,54,55]. This supports the need for constant monitoring of molds and mycotoxin content in animal feed.

## 4. Experimental Section

### 4.1. Samples

A total of 28 representative samples (1–2 kg per sample) of two different brands was collected from three hatcheries in the provinces of Río Negro and Neuquén (Argentina). Samples correspond to different fish life cycle stages: starter (13 samples), grower (13 samples: 4 pigmented and 9 unpigmented) and finisher (2 pigmented samples).

Samples were processed as follows: they were homogenized to obtain 1 kg of sample that was milled afterwards. Feed samples used for mycological analysis were processed upon arrival or stored in paper bags for no longer than 3 days at room temperature (about 25 °C). Long-term storage was done at −20 °C.

Table 5 shows the main ingredient (I1–I8) compounds present in commercial rainbow trout feeds.

All components present in the final product are listed in decreasing percentage order.

**Table 5:** Major ingredient declaration of compounds in commercial rainbow trout feeds.

Sample	Pellet Size (mm)	I1	I2	I3	I4	I5	I6	I7	I8
1	1.2	A	B	C	D	E	F	G	H
2	6.5	H	I	J	A	D	E	B	F
3	8.5	H	I	J	A	D	E	B	F
4	4.5	H	I	J	A	D	E	B	F
5	4.5	H	I	J	A	D	E	B	F
6	2.2–2.8	H	I	J	A	D	E	B	F
7	6.5	A	B	C	D	E	F	G	H
8	1.5–2.2	H	I	J	A	D	E	B	F
9	6.5	H	I	J	A	D	E	B	F
10	0.7	A	B	C	D	E	F	G	H
11	8	A	B	C	D	E	F	G	H
12	0.3–0.6	A	B	C	D	E	F	G	H
13	0.5–1.2	A	B	C	D	E	F	G	H
14	3.5	A	H	-	-	-	-	-	-
15	4.5	A	B	C	D	E	F	G	H
16	6.5	A	B	C	D	E	F	G	H
17	8.5	A	B	C	D	E	F	G	H
18	1.0–1.5	A	-	-	-	-	-	-	-
19	1.5–2.2	H	I	J	A	D	E	B	F
20	2.2–2.8	H	I	J	A	D	E	B	F
21	0.5–1.2	A	H	-	-	-	-	-	-
22	1.0–1.5	A	-	-	-	-	-	-	-
23	1.5–2.2	A	H	-	-	-	-	-	-
24	2.2–2.8	A	H	-	-	-	-	-	-
25	4.5	A	B	C	D	E	F	G	H
26	8	A	B	C	D	E	F	G	H
27	8	A	B	C	D	E	F	G	H
28	8	A	B	C	D	E	F	G	H

A: soybean expeller; B: disabled soybean; C: corn; D: wheat; E: wheat bran; F: corn gluten meal; G: soybean oil; H: fish meal; I: fish oil; J: chicken meal; -: not declared.

### 4.2. Mycobiota Analysis

Enumeration and isolation of fungi were performed using the dilute plate technique [46]. This procedure consists of mixing 10 g of each sample with 90 mL 0.1% peptone solution and then shaking for 20 minutes. Then, 0.1 mL of a spore suspension dilution were inoculated onto three different culture media. For total culturable fungi enumeration, Dichloran Rose Bengal Chloramphenicol Agar (DRBC) (Dichloran: Sigma-Aldrich, Intl., Buenos Aires, Argentina; Rose Bengal: Cicarelli, San Lorenzo, Argentina; Chloramphenicol: Calbiochem, Intl., Buenos Aires, Argentina; Agar: Britania, Buenos Aires, Argentina) was used. For xerophilic fungi enumeration, Dichloran 18% Glycerol Agar (DG18) (Glycerol: Biopack, Buenos Aires, Argentina). Additionally, for the selective isolation of *Alternaria* and *Fusarium* species, Dichloran Chloramphenicol Peptone Agar (DCPA) (Peptone: Britania, Buenos Aires, Argentina) was used. Incubation time for these plates was 7 days at 25 °C. On the other hand, a 12 h of light/12 h of darkness incubation photoperiod was used for DCPA plates.

Plates with colonies ranging from 10 to 100 were used for counting, and the results were expressed as colony-forming units per gram of sample (CFU/g) [46]. Individual CFU/g counts for each different colony type were recorded. Mold colonies suspected to belong to the *Alternaria* or *Fusarium* genera were cultured again onto plates containing water agar (WT). Additionally, molds suspected to belong to the *Aspergillus, Eurotium, Penicillium* and other genera were cultured on malt extract agar (MEA). Genus level identification of filamentous fungi was performed in accordance with Samson *et al*. [56].

Fungal isolates were identified at the species level according to the leading authorities: *Penicillium* sp. and *Aspergillus* sp. according to Pitt and Hocking [46]; *Fusarium* sp. according to Nelson *et al.* [57]; *Alternaria* sp. according to Simmons [58]; and other fungi according to Pitt and Hocking [46]. The isolation frequency (Fr) and relative density (RD) of genus/species were calculated according to Saleemi *et al.* [59] as follows:

Fr (%) = number of samples with a genus or species/total number of samples × 100

RD (%) = number of isolates of a genus or species/total number of fungi isolated × 100

All of the isolates were preserved on agar slants of MEA or potato dextrose agar (PDA) for *Alternaria* and *Fusarium* at 4 °C and cryopreserved in 18% glycerol at −20 °C.

### 4.3. Mycotoxin Analysis

To evaluate mycotoxin occurrence, trout feed samples were subjected to quantitative analyses using ELISA-based analytical test kits for aflatoxin, ochratoxin A, T-2 toxin, fumonisin, deoxynivalenol and zearalenone (RIDASCREEN FAST, R-Biopharm AG, Darmstadt, Germany). The extraction procedures were according to the manufacturer’s protocol. In brief, 5 g of each ground sample were extracted with 25 mL of 70% methanol for total aflatoxins, T-2 toxin, ZEA and total fumonisins. For OTA and DON, samples were extracted with 12.5 mL of 70% methanol or 100 mL of distilled water, respectively. Afterwards, samples were shaken vigorously for 3 minutes and the extracts filtered through Whatman N°1 paper (Whatman Inc., New Jersey, NY, USA). Then, aflatoxins, OTA, T-2 toxin and ZEA filtrates were diluted with distilled water in the ratio of 1:1 and fumonisin filtrates in the ratio of 1:14. Fifty microliters of the diluted filtrate per well were used for testing [9,10]. Analytical-grade methanol (Biopack, Buenos Aires, Argentina) was used for extractions. All reagents used for mycotoxin determinations were provided with the RIDASCREEN test kit. Each kit contains a microtiter plate coated with capture antibodies, toxins standard solutions, peroxidase conjugate and an anti-mycotoxin antibody for each mycotoxin, substrate/chromogen stained red, washing buffer and stop solution 1 N sulfuric acid.

Microtiter plate spectrophotometer (R-Biopharm AG, Darmstadt, Germany) was used for quantification, and absorbance was measure at 450 nm. Then, the logit/log function from the RIDA SOFT Win software (R-Biopharm AG, Darmstadt, Germany) was used for data evaluation.

### 4.4. Statistical Analysis

For analytical purposes, mycotoxin levels below the limits of detection (LOD) were calculated with RIDA SOFT Win software. These values below LOD (often referred to as “censoring”), but above LOD/√2, were considered for statistical analysis [47,60], since they provide meaningful information at the expense of a slightly higher measurement error. Undetected levels and values below LOD/√2 were assumed to be zero. Furthermore, since there was less than 60% of censored values among the data, the described replacement method is considered suitable [47,60,61].

Fungal counts and toxins content were analyzed with the Kruskal-Wallis test due to the rejection of the null hypothesis on the distribution normality with the Shapiro-Wilks test. The multiple range test for variables was employed to compare means of fungal counts and toxin content of samples from different provinces and growth stages. Differences were considered significant when *p* < 0.05. Spearman correlation coefficients were used to evaluate correlations between mycotoxins. Statistical significance was set at *p* < 0.001. InfoStat Version 2013 was used for all analyses.

## 5. Conclusions

There are few studies about mycotoxigenic fungi and the natural occurrence of mycotoxins in commercial fish feed, and even less in rainbow trout finished feeds. Therefore, due to the potential risk of contamination of this kind of feed by fungi and mycotoxins, regular monitoring is highly recommended. This is the first report on mycobiota contamination and the co-occurrence of several mycotoxins in rainbow trout feed in Argentina.
